# ADP-Ribosylation Factor 6 Mediates E-Cadherin Recovery by Chemical Chaperones

**DOI:** 10.1371/journal.pone.0023188

**Published:** 2011-08-10

**Authors:** Joana Figueiredo, Joana Simões-Correia, Ola Söderberg, Gianpaolo Suriano, Raquel Seruca

**Affiliations:** 1 Institute of Molecular Pathology and Immunology of the University of Porto, Porto, Portugal; 2 Medical Faculty of the University of Porto, Porto, Portugal; 3 Centre of Ophthalmology and Vision Sciences – Institute of Biomedical Research in Light and Image, Coimbra, Portugal; 4 Department of Genetics and Pathology, Uppsala University, Uppsala, Sweden; Stanford University, United States of America

## Abstract

E-cadherin plays a powerful tumor suppressor role. Germline E-cadherin mutations justify 30% of Hereditary Diffuse Gastric Cancer (HDGC) and missense mutations are found in 30% of these families. We found possible to restore *in vitro* mutant E-cadherin associated to HDGC syndrome by using Chemical Chaperones (CCs). Herein, our aim was to disclose the molecular mechanisms underlying the CCs effects in E-cadherin regulation. Using cells stably expressing WT E-cadherin or two HDGC-associated missense mutations, we show that upon DMSO treatment, not only mutant E-cadherin is restored and stabilized at the plasma membrane (PM), but also Arf6 and PIPKIγ expressions are altered. We show that modulation of Arf6 expression partially mimics the effect of CCs, suggesting that the cellular effects observed upon CCs treatment are mediated by Arf6. Further, we show that E-cadherin expression recovery is specifically linked to Arf6 due to its role on endocytosis and recycling pathways. Finally, we demonstrated that, as DMSO, several others CCs are able to modulate the trafficking machinery through an Arf6 dependent mechanism. Interestingly, the more effective compounds in E-cadherin recovery to PM are those that simultaneously inhibit Arf6 and stimulate PIPKIγ expression and binding to E-cadherin. Here, we present the first evidence of a direct influence of CCs in cellular trafficking machinery and we show that this effect is of crucial importance in the context of juxtamembrane E-cadherin missense mutations associated to HDGC. We propose that this influence should be taken into account when exploring the therapeutic potential of this type of chemicals in genetic diseases associated to protein-misfolding.

## Introduction

Cadherins are a family of adhesion molecules that play pivotal roles in tissue patterning during development and tissue architecture in adult. E-cadherin is a prototypical member of the classic cadherin family and is the best characterized cadherin, being the major component of the Adherens Junctions (AJs). The extracellular domain of E-cadherin binds Ca^2+^ and forms complexes with the extracellular domains of other E-cadherin molecules on neighboring cells [1], [2], [3]. The cytoplasmic domain of E-cadherin interacts with the actin cytoskeleton through a complex of anchoring proteins including α-, β-, p120- and γ-catenins [1], [4]. In normal tissues, E-cadherin plays a powerful tumor suppressor role and the assembly and maintenance of cadherin-catenins interactions is tightly regulated [2], [5].

E-cadherin expression is partially or completely loss in various types of cancer [6], [7] and this is associated to increased cell invasion and metastatic potential [8], [9]. In sporadic diffuse gastric cancer and lobular breast cancer, E-cadherin loss is associated with somatic mutations, loss of heterozygosity, promoter hypermethylation, aberrant glycosylation and overexpression of transcriptional repressors [10], [11], [12], [13], [14], [15], but these mechanisms explain only a rather limited percentage of cases with loss of E-cadherin.

More recently, post-transcriptional mechanisms such as endocytic and exocytic trafficking of E-cadherin have been described to be involved in E-cadherin regulation at the plasma membrane (PM) [16], [17], [18], [19]. Recent studies demonstrate that mature AJs are dynamic at the cell surface, following a continuous turnover through the activity of the cellular vesicle transport machinery, even in cells that do not appear to be moving [16], [17], [18], [19]. Thus, the redistribution of E-cadherin, spatially regulated by endocytosis and exocytosis, contributes to cell adhesion, cell polarity and cell rearrangement.

We have previously used cell lines stably expressing two distinct E-cadherin germline missense mutations found in the context of carriers of Hereditary Diffuse Gastric Cancer to map the key domain E-cadherin for its stable expression at PM [20]. Both mutants (R749W and E757K) are localized at E-cadherin juxtamembrane intracellular domain and lead to a significant decrease of E-cadherin membrane expression, impairing E-cadherin mediated cell-adhesion and inducing cell invasion [20]. However, we were able to relocate both mutant E-cadherin proteins to the PM by treating mutant expressing cells with Chemical Chaperones (CC), suggesting an interesting therapeutic possibility. Using CC, besides fully restoring E-cadherin PM expression, we were also able to rescue E-cadherin functionality for one of the mutants. Nevertheless, the mechanism involved in CC-dependent mutant E-cadherin rescue was not identified at that time.

In the present work, we aim to disclose the mechanism responsible for mutant E-cadherin expression rescue to the PM by the action of CCs. We hypothesize that CCs may act through the modulation of proteins involved in E-cadherin regulation and cellular trafficking, and focused our attention on ADP-ribosylation factor 6 (Arf6). Arf6 GTPase activation has been shown to promote E-cadherin endocytosis and induce invasion, by interacting and recruiting several other trafficking partners from the cytosol to the PM, namely Nm23-H1 and Gep100 [21], [22], [23].

To test our hypothesis we used the previous model system (E-cadherin negative cell lines lines stably transduced with the E-cadherin WT or missense mutations - R749W and E757K) treated with distinct CC and modulated the expression of Arf6, by RNAi or overexpression of Arf6 mutants. We show that upon CCs treatment, these cell lines have a decrease of Arf6 expression at the RNA and protein level. Additionally, we demonstrate that modulation of Arf6 expression partially mimics the effect of CCs on mutant E-cadherin recovery to the PM. Both results support the hypothesis that CC interfere with E-cadherin localization and function through the regulation of particular trafficking proteins. Furthermore, we found that DMSO is the most efficient CC in E-cadherin recovery to PM by simultaneously inhibiting Arf6 and stimulating Type Iγ phosphatidylinositol phosphate kinase (PIPKIγ) expression and binding to E-cadherin, supporting E-cadherin exocytosis and stabilization at the basolateral PM.

All our findings demonstrate that the cellular effects obtained upon CCs treatment are mediated by the modulation of the trafficking machinery, responsible for E-cadherin stability and turnover.

## Results

### DMSO rescues E-cadherin and modulates Arf6 expression

In order to understand the DMSO-dependent mechanism responsible for the rescue of mutant E-cadherin to the PM, we treated cells expressing wild type (WT) or R749W and E757K E-cadherin mutants with DMSO and analyzed the pattern of Arf6 expression.

**Figure 1:**
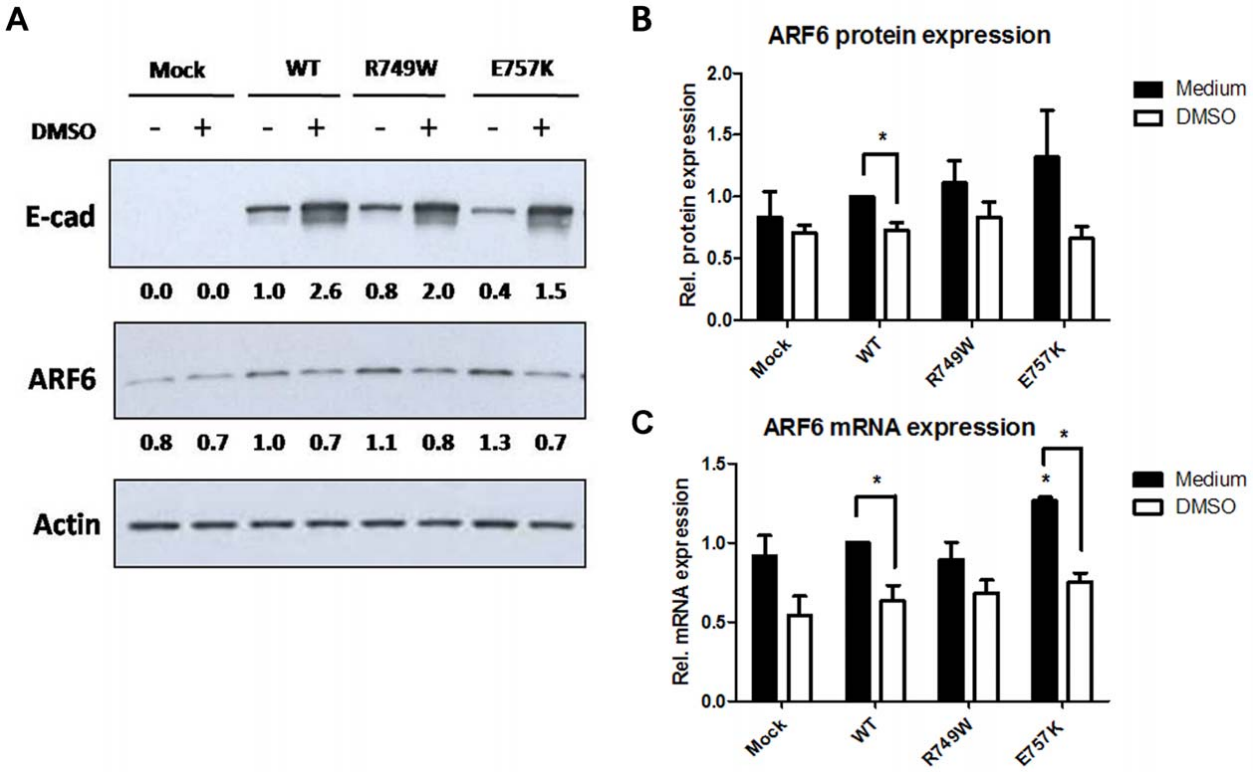
DMSO increases E-cadherin expression and modulates Arf6 expression. (A) CHO cells stably transduced with the empty vector (Mock) or with WT, R749W or E757K hEcadherin were treated with 2% DMSO for 24 h. E-cadherin, Arf6 and Actin were detected in whole cell lysates by Western Blot. Actin was used as a loading control. The images shown are representative of three independent experiments. The intensity of the bands was quantified and normalized against the non-treated WT E-cadherin expressing cells. The intensity average is shown below the respective images. (B) The graph shows the average + SE of Arf6 protein level in cells with or without DMSO treatment, in three independent experiments. (C) ARF6 and 18S mRNA levels were analyzed by real-time PCR. 18S was used as endogenous control. The graph represents the average + SE of ARF6 mRNA, n = 3 (* represents p≤0.05).

In this study we confirmed that E-cadherin expression is strongly increased upon DMSO treatment – 2.6 fold increase in the WT context, from 0.8 to 2.0 (2.5 fold) in the mutant R749W and from 0.4 to 1.5 (3.75 fold) in the mutant E757K, Figure 1A – and we verified that DMSO treatment leads to decreased Arf6 expression (Figure 1A–C), both at the protein and RNA level.

Taken together, the above referred results could suggest that E-cadherin recovery by CCs is mediated by an endocytosis decline.

### Distinct Chemical Chaperones recover E-cadherin expression and modulate Arf6

In order to clarify if the effects on Arf6 were specific of the treatment with the chemical chaperone DMSO, or if other CCs shared the same mechanism, we treated the cell lines stably expressing WT or mutant forms of E-cadherin with different CCs, and analyzed total and surface expression of E-cadherin, together with Arf6 expression. The set of CCs used was similar to what previously used by our group [20]. Further, MG132 proteasome inhibitor was also tested.

**Figure 2:**
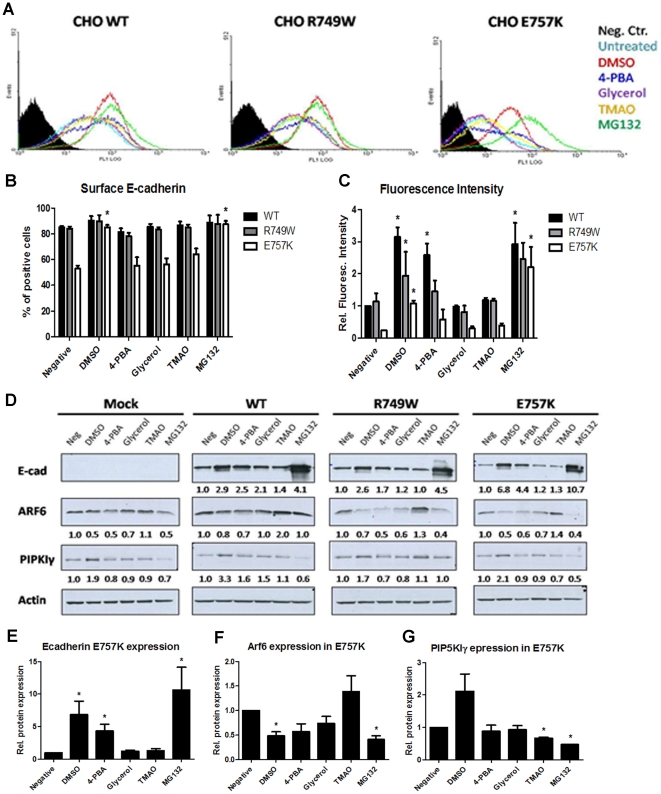
Distinct Chemical Chaperones recover E-cadherin expression by modulation of Arf6. CHO cells stably transduced with the empty vector (Mock) or with WT, R749W or E757K hEcadherin were treated with 2% DMSO, 5 mM 4-PBA, 5% Glycerol, 100 mM TMAO and 10μM MG132. (A) Flow cytometry technique was used to assess E-cadherin in cell surface. Each histogram represents the surface E-cadherin expression in cells untreated, treated with the different CC or with proteasome inhibitor MG132. The black area in the histogram represents the cells that were not incubated with primary antibody, this sample was used as negative control. (B) For each sample, the mean of cells expressing surface E-cadherin was calculated. The graph shows the average + SE of three independent experiments. (C) The mean fluorescence intensity was also quantified and normalized for cells untreated expressing WT E-cadherin. The graph shows the average + SE, n = 3. (D) E-cadherin, Arf6, PIPKIγ and Actin were detected in whole cell lysates by Western Blot. Actin was used as a loading control. The intensity of the bands was quantified and normalized against the untreated cells. The average of three independent experiments is shown below the respective sample. (E) The graph shows the average + SE of E-cadherin protein level in mutant E757K cells after treatments. (F) The bars represent the average + SE of Arf6 protein level in mutant E757K cells after treatments. (G) Quantification of PIPKIγ protein level in mutant E757K cells after treatments. * represents p≤0.05.

Flow cytometry results (Figure 2A–C) showed that within the set of CCs used, the most effective in E-cadherin rescue to the PM is DMSO, followed by 4-PBA. Interestingly, these treatments, as well as MG132, not only led to an increased number of E-cadherin positive cells (Figure 2B) but also generated more intense staining (Figure 2C), meaning that more E-cadherin molecules are rescued to the PM. The increased number of positive cells was statistically significant for the E757K expressing cells treated with DMSO (p = 0.0004) or with MG132 (p = 0.0007) when compared with untreated cells. The fluorescence intensity was significantly increased upon treatment with DMSO (WT p = 0.002, R749W p = 0.032 and E757K p = 0.0011), 4-PBA (WT p = 0.012) and MG132 (WT p = 0.043 and E757K p = 0.036).

As shown in Figure 2D and 2E, total E-cadherin expression was increased upon DMSO, 4-PBA, Glycerol and MG132 treatments. The increase of total E-cadherin expression induced by CCs or by MG132 proteasome inhibitor is accompanied by the downregulation of Arf6 (Figure 2D–F). These results reveal that E-cadherin enhancement of expression is associated to Arf6 downregulation, independently of the CC used. Interestingly, we found that DMSO is the only CC able to simultaneously inhibit Arf6 and stimulate PIPKIγ expression (Figure 2D and 2G) and PIPKIγ is described to be an important partner involved in E-cadherin exocytosis and transport to PM [16], [24].

The proteasome inhibition by MG132 generate the highest increase of E-cadherin total expression, which is also reflected in the PM, but also leads to Arf6 downregulation in the mutant E-cadherin expressing cells. In this context, the decrease of Arf6 expression could be the result of the degradation machinery blockage by MG132 – indicating the involvement of Arf6 in degradation pathways – and, as consequence, it leads to mutant E-cadherin accumulation.

### Arf6 inhibition leads to an increase of WT and mutant E-cadherin expression

**Figure 3:**
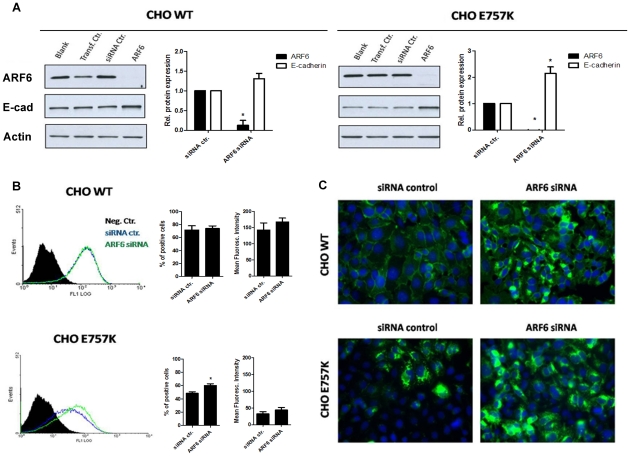
Arf6 specific inhibition by siRNA leads to an increase of E-cadherin protein expression. Arf6 inhibition by siRNA was performed in CHO cells stably transduced with WT or E757K hEcadherin. (A) Cell lysates were extracted 48 h after transfection. E-cadherin, Arf6 and Actin were analyzed by Western Blot. Actin was used as a loading control. The intensity of the bands was quantified and normalized against the siRNA control. In the graph, bars represent the average + SE of E-cadherin or Arf6 protein expression of three independent experiments. (B) Flow cytometry technique was used to assess E-cadherin cell surface expression. Each histogram represents the cell surface expression of E-cadherin in WT or E757K cells treated with siRNA control (blue) or with specific siRNA for Arf6 (green). The black area in the histogram represents the cells that were not incubated with primary antibody; this sample was used as negative control. For each sample, the number of cells expressing surface E-cadherin as well as the mean fluorescence intensity (arbitrary units) was calculated. The graphs show the average + SE of three independent experiments (* represents p≤0.05). (C) Cells were fixed 48 h after transfection and immunostained with anti-human E-cadherin antibody. Nucleus was counterstained with DAPI. The pictures were taken under a 40× objective.

To understand the relevance of Arf6 downregulation in the process of E-cadherin recovery (WT and mutant) to the PM, we specifically inhibited Arf6 expression by siRNA on cells stably transduced with WT or mutant E-cadherin [20]. The inhibition of Arf6 was validated by western blot and the efficiency was maximum (up to 90%) in all tested cell lines at 48 h, using 50 nM of siRNA (Figure 3A).

We observed that knockdown of Arf6 causes an increase of E-cadherin protein levels in cells expressing WT or mutant E-cadherin. The level of E-cadherin protein recovery was significantly more pronounced in the mutant E757K (p = 0.045) (Figure 3A).

We used flow cytometry (FCM) to test whether the increase in E-cadherin protein levels promoted by Arf6 silencing reflects an increase in E-cadherin localized at the PM. We show that Arf6 inhibition does not alter the number of cells expressing surface E-cadherin in WT cells, while for mutant E757K it induces a significant (p = 0.035) increase in cells positive for E-cadherin at the PM (Figure 3B). We believe that this difference between mutant and WT background may be related to the initial amount of surface E-cadherin. Accordingly, in cells transduced with WT E-cadherin, the consequences of inhibiting Arf6-dependent endocytosis are milder due to the initial WT overload at the PM.

Further, we have analyzed E-cadherin localization upon Arf6 siRNA treatment using immunocytochemistry (Figure 3C) and show that Arf6 silencing induces also accumulation of the protein in the cytoplasm in both WT and mutant background. This effect resembles the results obtained previously upon proteasome inhibition as a consequence of Endoplasmic Reticulum Associated Degradation (ERAD) [20].

### Effects of Arf6 activation or inactivation in the regulation of mutant E-cadherin

Our results suggest that Arf6 downregulation is involved in the rescue of mutant E-cadherin to the PM. Accordingly, we decided to dissect the functionality of Arf6 in our model. To this end, we transiently transfected stable E-cadherin expressing cells with vectors expressing mutant forms of Arf6: ARF6 Q67L (constitutive active form) and ARF6 T27N (dominant negative form). These vectors are well characterized and have been widely used to elucidate the role of Arf6 in several studies [23], [25], [26], [27].

**Figure 4:**
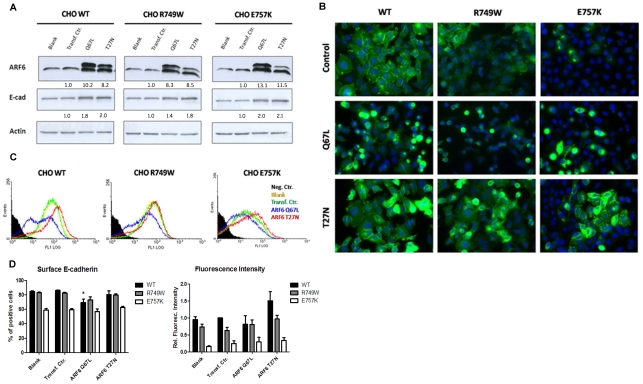
Arf6 activation or inactivation interferes with E-cadherin expression and localization. CHO WT, CHO R749W and CHO E757K were transiently transfected with vectors expressing mutant forms of Arf6: ARF6 Q67L (constitutive active form) and ARF6 T27N (dominant negative form). (A) Arf6, E-cadherin and Actin were detected in whole cell lysates by Western Blot. Actin was used as a loading control. The intensity of the bands was quantified and normalized against the transfection control. The intensity average of three independent experiments is shown below the respective sample. (B) Cells were fixed and immunostained with anti-human E-cadherin antibody. Nucleus was counterstained with DAPI. The pictures were taken under a 40× objective. (C) Flow cytometry technique was used to assess E-cadherin cell surface expression. Each histogram represents surface E-cadherin in cells untransfected (yellow), transfected with ARF6 Q67L (blue) or with ARF6 T27N (red) and the transfection control (green). The black area in the histogram represents the cells that were not incubated with primary antibody; this sample was used as negative control. The results are representative of three independent experiments. (D) For each sample, the number of cells expressing surface E-cadherin was calculated. The mean fluorescence intensity was also quantified and normalized against the transfection control of WT expressing cells. The graphs show the average + SE, n = 3 (* represents p≤0.05).

Different time points were tested for analysis (data not shown) and 24 h post-transfection was chosen, when a stronger effect with less cell toxicity was observed. After transfection, cells were either lysed, prepared for flow cytometry or for immunofluorescence. Analysis of protein expression by western blot demonstrated that the transfection procedure was efficient. As shown in Figure 4A, cells transfected with the two vectors express higher levels of Arf6 than untransfected cells (ARF6 Q67L: 10.2 fold increase in the WT context, and 8.3 and 13.1 in the mutants R749W and E757K, respectively; ARF6 T27N: 8.2 fold increase in WT, 8.5 in R749W and 11.5 in E757K). The two bands present in the transfected conditions correspond to the endogenous Arf6 (bottom band) and the hemagglutinin (HA)-tagged Arf6 (the top band). As observed by Palacios F. and colleagues, the transfection of artificial mutant forms of Arf6 produced an effect on Arf6 endogenous expression [23]. It seems that cells have an autoregulation mechanism for Arf6.

The expression of constitutive active form of Arf6, defective in GTP binding and hydrolysis (ARF6 Q67L), showed a strong effect in the expression of E-cadherin WT, R749W or E757K. Upon this transfection, cells lose cell-cell contacts and the epithelial phenotype, becoming isolated and with round-like morphology (Figure 4B). In E-cadherin WT expressing cells, FCM results (Figure 4C–D) show that constitutive active Arf6 leads to a statistically significant E-cadherin delocalization from PM (17% decrease, p = 0.029). Immunofluorescence results show that this decrease in PM is accompanied by E-cadherin cytoplasmic accumulation (Figure 4B). We also observe a significant (p = 0.033) increase in total E-cadherin expression (Figure 4A) and this effect is probably due to the continuous *de novo* synthesis of E-cadherin. To test this hypothesis, we inhibited protein synthesis using Cyclohexamide but this treatment also blocked the synthesis of ARF6 Q67L, not allowing to demonstrate our assumption (data not shown).

For the mutants R749W or E757K, the Arf6 Q67L transfection also produced decreased PM expression and intracellular accumulation. However, this effect is less evident for mutant E-cadherin as a consequence of lower amount of E-cadherin in the cell due to the constitutive premature degradation of the mutant forms as previously described [20].

The dominant negative, GDP-bound, mutant of Arf6 (ARF6 T27N) generates increased levels of E-cadherin expression in the different cell lines, as shown by western blot (Figure 4A), similarly to the results observed upon Arf6 silencing by siRNA. Flow cytometry demonstrates that the dominant negative Arf6 produces a slight increase (3.2%) in the number of cells expressing mutant E757K E-cadherin at the cell surface and a more intense staining in WT and in mutant R749W cells, from 1.0 to 1.5 fold increase in the WT context and from 0.6 to 1.0 fold increase in the mutant R749W (Figure 4C–D). Moreover, and similarly to what was verified by siRNA, the ARF6 T27N also results in intracellular accumulation of E-cadherin in cells expressing either WT or mutant E-cadherin (Figure 4B) and this might be the result of the failure of Arf6 role in recycling [27], [28]. As expected, the effect of this non-functional Arf6 is less evident than that of the siRNA-mediated knockdown, due to the presence of endogenous, functional, Arf6.

These results confirm the involvement of Arf6 in E-cadherin trafficking and regulation and show that manipulation of Arf6 could be used to restore E-cadherin. Accordingly, in cells with normal E-cadherin expression, the constitutive activation of Arf6 induces E-cadherin internalization and consequently cells loose the epithelial phenotype. By contrast, in E-cadherin mutant context with decreased PM expression, the inactivation of Arf6 induces E-cadherin rescue at the plasma membrane.

### Pharmacological inhibition of endocytosis promotes E-cadherin increase at the plasma membrane

**Figure 5:**
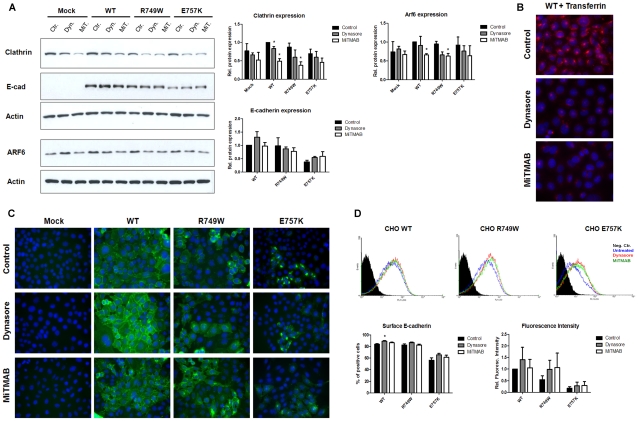
Endocytosis inhibition increases E-cadherin at the plasma membrane. CHO cells stably transduced with the empty vector (Mock) or with WT, R749W or E757K hEcadherin were treated with Dynasore or MiTMAB for 17h. (A) Clathrin, E-cadherin and Arf6 expression were analyzed in whole cell lysates by Western Blot. Actin was used as a loading control. The intensity of the bands was quantified and normalized against the untreated WT cells. The graphs show the average + SE of protein level, in three independent experiments. (B) After treatment, Transferrin 594 was added to cells. Nucleus was counterstained with DAPI. The pictures were taken under a 63× objective. (C) Cells were fixed and immunostained with anti-human E-cadherin antibody. Nucleus was counterstained with DAPI. The pictures were taken under a 40× objective. (D) Flow cytometry technique was used to assess E-cadherin cell surface expression. Each histogram represents the cell surface expression of E-cadherin in cells WT, R749W or E757K, treated with Dynasore (red) or MiTMAB (green) or untreated (blue). The black area in the histogram represents the cells that were not incubated with primary antibody, this sample was used as negative control. For each sample, the number of cells expressing surface E-cadherin was calculated. The mean fluorescence intensity was also quantified and normalized against the control of WT expressing cells. The graphs show the average + SE, n = 3 (* represents p≤0.05).

Knowing that Arf6 has a role in the endocytic process but also in recycling pathways, we investigated whether the increase of WT and mutant E-cadherin expression was a specific feature of endocytosis inhibition or an effect related to the inhibition of E-cadherin recycling. To answer this question we treated the cell lines stably expressing WT or the mutant forms of E-cadherin with two pharmacological inhibitors of endocytosis, Dynasore and MiTMAB. To control the efficiency of the endocytic process blockage by these inhibitors, we tested the internalization of Transferrin conjugates. The Transferrin uptake is the approach commonly used to assess the endocytosis inhibition [29], [30]. In addition, we analyzed the expression of Clathrin heavy chain, the main structural protein of clathrin-coated pits [31], as it was described that Dynasore reduces the clathrin coated pit formation [29]. We could confirm the action of the inhibitors by a decrease in Clathrin protein levels (Figure 5A), concomitant with a decrease of Transferrin594 internalization (Figure 5B).

After pharmacological inhibition of endocytosis, we evaluated E-cadherin total protein level by western blot. In the three cell lines, Dynasore or MiTMAB did not promote significant alterations in E-cadherin level, however Arf6 protein is reduced confirming its involvement in the endocytic pathway (Figure 5A).

Under the same treatment conditions, E-cadherin subcellular localization and surface expression were further evaluated by immunofluorescence staining and FCM (Figure 5C–D). We verified that the number of cells expressing E-cadherin in the PM is increased by Dynasore: 5.1% in WT (p = 0.035), 4.3% in R749W and 9.0% in E757K cells; and by MITMAB: 2.6%, 0.1% and 4.8%, respectively (Figure 5D). The mean fluorescence intensity was also increased with the endocytosis blockage by Dynasore or MITMAB, although the differences were not statistically significant (Figure 5D). Importantly, the endocytosis inhibition does not induce an abnormal E-cadherin accumulation in the cytoplasm of the cells, as happens with the specific modulation of Arf6 by siRNA or by Arf6 mutant constructs.

### DMSO improves E-cadherin interaction with PIPKIγ, β- and p120-catenins leading to increased E-cadherin exocytosis and stability

**Figure 6:**
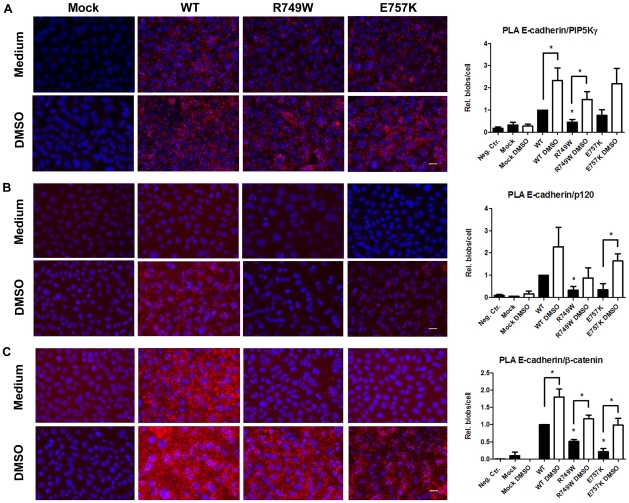
DMSO improves E-cadherin interaction with PIPKIγ, β- and p120-catenins. CHO cells transduced with the empty vector (Mock) or with WT, R749W or E757K hEcadherin were treated with 2% DMSO or with Medium, and the interaction of E-cadherin with PIPKIγ (A), p120- (B) and β-catenins (C) was assessed by PLA. Cells were fixed and incubated with antibodies against E-cadherin and PIPKIγ or E-cadherin and p120 or E-cadherin and β-catenin. In the negative control, CHO WT cells were incubated only with the antibody against E-cadherin. Close proximity of oligonucleotide-ligated secondary antibodies allows a rolling-circle amplification and the detection of the rolling-circle amplification product by a fluorescently labelled probe. Nuclei were counterstained with DAPI. The pictures were taken under a 40× objective. The number of spots per cell was quantified in each condition. The graphs show the average of relative number of blobs per cell + SE, n = 3 (* represents p≤0.05).

In order to test whether DMSO also promotes E-cadherin exocytosis and stability at PM, we tested if cells upon DMSO treatment exhibited an increased interaction between E-cadherin and PIPKIγ, justifying the high efficiency of DMSO in E-cadherin recovery to the PM. It is well known that PIPKIγ binds directly to E-cadherin in a homeostatic situation [24]. Using Proximity Ligation Assay (PLA), we could verify that, under normal conditions, the mutants R749W and E757K interact less with PIPKIγ than WT E-cadherin, 0.5 and 0.8 respectively, and this decrease is statistically significant for the mutant R749W (p = 0.005), as showed in the Figure 6A. However, it is possible to significantly improve this interaction with DMSO treatment: 2.3 fold for WT (p = 0.05), from 0.5 to 1.5 (3.0 fold, p = 0.03) for the mutant R749W and from 0.8 to 2.2 (2.75 fold) for the mutant E757K.

Because p120- and β-catenins link directly to E-cadherin [1], [4], [16], [17], [19], [32] and cadherin-catenins interaction is essential to E-cadherin transport and stabilization on plasma membrane and consequently affect E-cadherin function, we analyzed these interactions in the same context: medium or DMSO treatment. The PLA results show that, without treatment, both E-cadherin mutants are significantly less able to interact with p120-catenin (Figure 6B; p = 0.017 for the mutant R749W) and β-catenin (Figure 6C; p = 0.0005 for the mutant R749W and p = 0.001 for the mutant E757K). However, DMSO strongly increases its binding ability (Figure 6B–C). WT E-cadherin increases its interaction with p120 4.2 fold and with β-catenin 1.8 fold (p = 0.025). In the case of R749W E-cadherin, its interaction with p120 increases from 0.3 to 0.9 and with β-catenin from 0.5 to 1.2 (p = 0.006). For the mutant E757K, the relation with p120 improves from 0.4 to 1.6 (p = 0.04) and with β-catenin from 0.2 to 1.0 (p = 0.02).

## Discussion

Germline mutations of the CDH1 gene result in loss of function of E-cadherin gene in approximately one third of the cases of Hereditary Diffuse Gastric Cancer (HDGC). Most of the germline CDH1 mutations identified to date are of the nonsense type, leading to alternative premature termination codons. They are commonly downregulated by non-sense mediated decay [33], a mechanism of mRNA surveillance, making their pathogenic significance easy to infer. However, germline missense mutations occur in about 30% of the families [34], and in contrast to truncating mutations, their pathogenicity is not straight-forward, therefore constituting a problem in terms of genetic counseling. To circumvent this limitation and improve the genetic counseling, we have established an *in vitro* cellular model to classify the significance of missense CDH1 mutations [35]. Within all the mutants functionally tested [20], [35], [36], [37], [38], [39], [40], [41], the juxtamembrane domain missense E-cadherin mutants R749W and E757K, besides showing impaired E-cadherin dependent adhesion and increased invasiveness, also display reduced total and surface protein expression, 60% and 25% respectively, due to ERAD [20]. Interestingly, in our previous study, we showed that CC DMSO was able to recover mutant E-cadherin expression to the PM and function but the molecular mechanism underlying this effect remained to be determined [20].

In the present work, we focused on the effect of the CC on Arf6, a key protein involved in E-cadherin regulation and cellular trafficking [21], [23], [42]. We have found that DMSO treatment rescues E-cadherin expression and at the same time inhibits Arf6 expression at the RNA and protein level (Figure 1). These findings suggest that the stabilization of E-cadherin in the PM, in response to CC treatment, could be mediated by a decrease of the endocytic pathway, resulting from Arf6 downregulation.

To understand the significance of Arf6 downregulation in this background, we performed specific inhibition of Arf6 in cells stably transduced with WT or mutant E-cadherin. The knockdown of Arf6 by siRNA leads to increased total E-cadherin expression, both in the WT and mutant background, with a significant increase observed for the mutant E757K. Furthermore, the quantity of E-cadherin molecules present in the cell surface is improved, independently of E-cadherin context. The observations that Arf6 silencing leads to increased total E-cadherin expression and cytoplasmic accumulation of E-cadherin protein (Figure 3), suggests that Arf6 silencing interferes with the process of E-cadherin recycling and degradation. Our findings are in accordance with the role previously described for Arf6, regarding endosomes recycling and protein degradation [27], [28]. In 1995, the work of D'Souza-Schorey and collaborators showed that, in CHO cells, expression of the dominant negative mutant, ARF6 T27N, resulted in intracellular distribution of transferrin receptors and an inhibition of transferrin recycling to the cell surface [25]. A large number of studies followed, corroborating that finding [26], [43], [44], [45]. More recently, evidence has emerged suggesting Arf6 as an indirect regulator of the proteasome. Arf6 promotes actin remodeling through its interaction with POR1 (partner of Rac1) and Arfaptin-2 [46], [47]. It was shown that Arfaptin-2 inhibits the function of 26S proteasome, and as such modulates some cellular processes that are dependent on proteasome function [48], [49].

To further dissect the regulation of mutant E-cadherin by Arf6, we have used Arf6 mutants defective in GTP hydrolysis (Q67L, constitutively active) and GDP binding (T27N, dominant negative). As expected, the constitutive active Arf6 (Q67L) promoted E-cadherin internalization from cell surface, resulting in decreased levels of E-cadherin at the PM, and cytoplasmic accumulation of E-cadherin protein (Figure 4). In contrast, expression of a dominant negative mutant (T27N) resulted in an increased PM expression and also intracellular accumulation of E-cadherin (Figure 4).

To clarify if the increase of total and surface E-cadherin, in response to Arf6 modulation, could be related to an effect of endocytosis inhibition or due to the blockage of E-cadherin recycling, we decided to perform endocytosis-inhibition assays using pharmacological inhibitors of the dynamin-dependent endocytic pathways, Dynasore and MiTMAB. These two compounds are potent Dynamin-GTPase activity inhibitors [29], [30]. Dynamin is a GTPase enzyme essential for vesicle formation and is required for membrane constriction and fission during endocytosis [29], [30], [50]. The major route of E-cadherin internalization is reported to be a Clathrin dependent route [50], [51], [52], [53], [54], although non-Clathrin-dependent pathways have been implicated, including Caveolae-mediated [55] and an EGF-induced macropinocytosis pathway [56]. Nevertheless, all mentioned pathways are dependent of Dynamin activity [50], [57]. Upon endocytosis inhibition, we observe that, independently of the E-cadherin background, E-cadherin level of expression is not significantly altered, although there is an increase in the number of cells expressing E-cadherin in PM as well as an increase in the number of E-cadherin molecules present in the PM (Figure 5). These results suggest that the E-cadherin increase in the PM, in response to Arf6 modulation, is a consequence of the endocytic pathway decrease but E-cadherin accumulation in the cytoplasm of the cells, is probably due to the inhibition of E-cadherin recycling and degradation.

Finally, we demonstrated that, together with DMSO, several other CCs are able to modulate Arf6 expression. Interestingly, the more effective compound in E-cadherin recovery to PM is the one that simultaneously inhibit Arf6 and stimulate PIPKIγ (Figure 2) showing that stabilization of E-cadherin at the cell surface is due to a decrease of the endocytic and recycling machinery and stimulation of exocytic pathways. It is known that the role of Arf6 at the cell surface is mediated by its effect on phospholipid metabolism [58], [59], [60] and it was shown that Arf6 regulates PIPKIγ [59]. Moreover it was demonstrated that PIPKIγ binds directly to E-cadherin, modulating its cellular trafficking [24]. PIPKIγ depletion or disruption of its binding to E-cadherin results in defects in E-cadherin transport and blocks AJs assembly [24]. Therefore, we tested whether the increase of PIPKIγ expression upon DMSO treatment could imply increased binding to E-cadherin and, in consequence, increased E-cadherin transport to the PM and stabilization at AJs. To verify the interaction between E-cadherin and PIPKIγ, we used PLA [61] and have shown that DMSO was able to improve the interplay of E-cadherin with PIPKIγ, β- and p120-catenins, which have crucial roles in E-cadherin exocytosis, transport and stability in the PM (Figure 6).

Proteasome inhibition by MG132 also results in E-cadherin increase and stabilization at the PM (Figure 2). It was already described that MG132 regulates the stability of E-cadherin at the PM through blocking its endocytosis induced by Transforming Growth Factor (TGF)-β, however the molecular mechanism implicated was unknown [62]. Our findings are not only in agreement with that study, but also show that MG132 effect is also mediated by downregulation of Arf6.

Together, our results show that mutant E-cadherin expression rescue by the CCs, is mediated by the modulation of E-cadherin trafficking partners, namely Arf6 and PIPKIγ. In this context, we propose that Arf6 downregulation results in the blockage of E-cadherin endocytosis, but also in its recycling to the PM and possibly its degradation blockage, leading to a significant cytoplasmic accumulation of the protein. Artificial manipulation of Arf6 shows that Arf6 inhibition is essential, but not sufficient, to completely restore the E-cadherin mutant associated to Hereditary Diffuse Gastric Cancer syndrome to the PM. In turn, PIPKIγ binding, as well as β- and p120-catenins, are responsible for the improvement of E-cadherin exocytosis, transport and stabilization in the PM.

Thus, by dissecting the mechanism of action of CCs, and its influence on E-cadherin trafficking regulators, we propose that mutant E-cadherin may be stabilized at the PM by inhibition of endocytosis and decreased recycling and degradation, but also increased exocytosis. Accordingly, we propose that the CCs effect is the result of a balance between the different trafficking processes: exocytosis, endocytosis, recycling and degradation.

With this work, we present the first evidence of a direct influence of CCs in the cellular trafficking machinery and we propose that this influence should be taken into account when exploring the therapeutic potential of these types of molecules. CCs have been already studied experimentally and reported to reverse and repair conformational defects of some mutated proteins [63]. Therefore CCs are promising therapies in cancer and in a large number of protein-misfolding diseases as Alzheimer's disease, Parkinson's disease, Huntington's disease, Creutzfeldt–Jakob disease, cystic fibrosis, nephrogenic diabetes insipidus, Gaucher's disease and many other degenerative and neurodegenerative disorders [63]. Nevertheless, the toxicity of these types of molecules is often problematic as therapy. The success of CCs in therapeutic approaches depends on our ability to understand their mechanism of action, enabling the alternative of manipulating the involved molecules in a viable way.

## Materials and Methods

### Cell Culture

CHO (Chinese Hamster Ovary) cells (ATCC number: CCL-61) were transduced with the following vectors: empty vector (Mock), WT hE-cad, R749W and E757K, as previously described by Simões-Correia *et al.* [20]. The transduced cells were selected by antibiotic resistance to blasticidin (5μg/ml). Cells were grown at 37°C under 5% CO_2_ humidified air, in α-MEM (+) medium (Gibco, Invitrogen) supplemented with 10% fetal bovine serum (HyClone, Perbio), 1% penicillin/streptomycin (Gibco, Invitrogen) and blasticidin (Gibco, Invitrogen).

### Cell Treatments

Cells were plated in 6-well plates and incubated with Chemical Chaperones for 24 h and with endocytosis inhibitors or proteasome inhibitor MG132 for 17 h. The CCs used were 2% dimethyl sulfoxide (DMSO; Sigma), 5 mM 4-phenylbutyric acid (4-PBA; Sigma), 5% glycerol (Sigma) and 100 mM trimethylamine-N-oxide (TMAO; Sigma). The endocytosis inhibitors used were Dynasore (80µM; Sigma) and MiTMAB (20µM; Calbiochem). The MG132 (Calbiochem) was used at 10μM concentration.

### Transfections

A set of 4 different siRNAs targeting ARF6 mRNA was purchased from Dharmacon and prepared according to the manufacturer's instructions. In parallel, nonsilencing siRNA duplexes (Dharmacon) were used as negative control. Before transfection, 60% confluent monolayers of CHO WT, CHO R749W or CHO E757K cells plated onto 6-well plates were washed with PBS and incubated in serum and antibiotic-free medium. Cells were transiently transfected with 0-150 nM siRNA, using Lipofectamine 2000 transfection reagent (Invitrogen). At the end of each transfection, putative cytotoxic effects were evaluated, analyzing cell viability. Efficiency of depletion was maximum at 48 h, and ARF6-07 and was chosen from the set of siRNAs and used at 50 nM.

For plasmid transient transfections, 1µg of DNA of each vector was used and the transfection procedure was the same as described above. The Q67L and T27N ARF6 constructs were kindly provided by D'Souza-Schorey C. (Department of Biological Sciences, University of Notre Dame, Notre Dame, USA).

### Flow cytometry (FCM)

Cells were grown to a confluent monolayer, detached with Versene (Gibco, Invitrogen) and resuspended in ice-cold PBS with 0.05 mg/ml CaCl_2_. For all conditions, 5×10^5^ cells were centrifuged for 5 min at 1500 rpm and 4°C and washed in PBS with 0.05 mg/ml CaCl_2_ and 3% BSA. Cells were incubated for 30 min with the extracellular primary antibody against E-cadherin, HECD1 (Zymed Laboratories) at 1∶50 dilution. Cells were washed twice and incubated with Alexa Fluor 488 goat anti-mouse (1∶250; Invitrogen) in the dark for 30 min. Finally, the cells were washed and resuspended in 0.5 ml of washing solution. At least 5×10^4^ cells were analyzed in a Coulter Epics XL-MCL flow cytometer. The data were analyzed with WinMDI software.

### Immunofluorescence staining

Cells were seeded on 6-weell plates on top of glass coverslips and grown to at least 80% confluence. Fixation was performed in ice-cold methanol for 20 min, followed by washing and blocking in 5% BSA in PBS for 30 min at room temperature. The mouse monoclonal E-cadherin antibody (BD Biosciences) was used at 1∶300 dilution in PBS with BSA 5% and incubated for 1 h at room temperature. An Alexa Fluor 488 goat anti-mouse (1∶500; Invitrogen) was applied for 1 h in dark as secondary antibody. The coverslips were mounted on slides using Vectashield with DAPI (Vector Laboratories). Images were acquired on a Carl Zeiss Apotome Axiovert 200 M Fluorescence Microscope using 20× and 40× objectives. Images were taken with an Axiocam HRm camera and processed with the Zeiss Axion Vision 4.8 software.

### Transferrin uptake

Cells were plated on glass coverslips and grown to at least 80% confluence. After cell treatment with Dynasore (80µM; Sigma) and MiTMAB (20µM; Calbiochem), as described before, 5µg/ml Transferrin 594 (Invitrogen) was added to each well and incubated for 15 min at 37°C. Fixation was performed in 4% formaldehyde in PBS for 30 min at 4°C followed by blocking of the aldehyde groups with 50 mM NH_4_Cl in PBS for 10 min at room temperature. Permeabilization was done with 0.2% Triton X-100 in PBS for 10 min. The coverslips were mounted on slides using Vectashield with DAPI (Vector Laboratories). Images were acquired on a Carl Zeiss Apotome Axiovert 200M Fluorescence Microscope using 40× and 63× objectives. Images were taken with an Axiocam HRm camera and processed with the Zeiss Axion Vision 4.8 software.

### Western blotting

Cells were lysed in cold Catenin lysis buffer – 1% Triton X-100 (Sigma), 1% Nonidet P-40 (Sigma) in PBS – enriched with a protease inhibitor cocktail (Roche) and a phosphatase inhibitor cocktail (Sigma). The proteins were quantified using a modified Bradford assay (Bio-Rad). For analysis of total protein samples, 25μg of proteins were eluted in sample buffer, and loaded in 7.5% or 12% SDS–PAGE, depending on the mass of the molecules. The proteins were then electroblotted onto a Hybond ECL membrane (Amersham Biosciences). Membranes were blocked with 5% non-fat milk or 4% BSA and 0.5% Tween-20 in PBS and immunoblotted with antibodies against E-cadherin (1∶1000, BD Biosciences), Arf6 (1∶100, Santa Cruz Biotechnology), PIP5K1C (1∶1000, Cell Signaling Technologies), Clathrin Heavy Chain (1∶1000, Cell Signaling Technologies) or Actin (1∶1000, Santa Cruz Biotechnology). Donkey anti-rabbit (Amersham Biosciences), sheep anti-mouse (Amersham Biosciences) or donkey anti-goat (Santa Cruz Biotechnology) HRP-conjugated secondary antibodies were used, followed by ECL detection (Amersham Biosciences). Immunoblots were quantified with the Quantity One Software (Bio-Rad).

### Real-Time PCR

Cells were grown to a confluent monolayer and total RNA was extracted with Tripure (Roche) according to the manufactureŕs protocol. cDNA was produced from 1μg of RNA with Superscript II Reverse Transcriptase (Invitrogen) and random hexamer primers (Invitrogen). Quantitative real time-PCR (qRT-PCR) was carried out in triplicates for the target ARF6 and for the endogenous control 18S using as probe sets Mm00500208_s1 and Hs99999901_s1 (Applied Biosystems), respectively. Data was analyzed by the absolute quantification method, applying a standard curve of serial dilutions for the target gene and endogenous control, in each plate, using an ABI Prism 7000 Sequence Detection System (Applied Biosystems).

### Proximity Ligation Assay

Cells seeded on top of glass coverslips were treated with DMSO, as previously described. After treatment, cells were fixed in ice-cold methanol for 20 min and subjected to PLA using Duolink Detection kit (Olink Bioscience, Uppsala, Sweden) according to the manufacturer's instructions for Duolink Blocking solution and Detection protocol. Briefly, slides were blocked, incubated with antibodies directed against E-cadherin (from BD Biosciences, mouse, 1∶100 or from Cell Signaling, rabbit, 1∶30), PIP5K1C (BD Biosciences, mouse, 1∶30), p120 (BD Biosciences, mouse, 1∶30) and β-catenin (Sigma, rabbit, 1∶100) and thereafter incubated with PLA probes, which are secondary antibodies (anti-mouse Minus and anti-rabbit Plus) conjugated to unique oligonucleotides. Amplification oligonucleotides were hybridized to probe pairs and circularized by ligation. The DNA circle was then amplified using rolling circle amplification into a bundle of single stranded DNA anchored to one of the antibodies and could be detected by addition of complementary fluorophore-labeled oligonucleotides. The coverslips were mounted on slides using Vectashield with DAPI (Vector Laboratories). Images were acquired on a Carl Zeiss Apotome Axiovert 200M Fluorescence Microscope using 20× and 40× objectives. Images were taken with an Axiocam HRm camera and processed with the Zeiss Axion Vision 4.8 software. The quantification of the dots was performed using BlobFinder V3.2.

### Statistical analysis

Two-tailed paired Student's *t*-test was used to perform statistical analysis. In all analysis *p*<0.05 was required for statistical significance. Statistical analysis was done using StatView software program (PC version).
